# Spontaneous Spine Fracture in Patient with Ankylosing Spondylitis under Spinal Anesthesia: A Case Report and Review of the Literature

**DOI:** 10.3390/medicina57101051

**Published:** 2021-10-01

**Authors:** Ul-Oh Jeung, Dae-Chang Joo, Sung-Kyu Kim, Chae-Jin Im

**Affiliations:** 1 Department of Orthopaedic Surgery, Sun Cheon Hana Hospital, Suncheon 57964, Korea; junguri994@naver.com (U.-O.J.); derwind3835@hanmail.net (D.-C.J.); 2 Department of Orthopaedic Surgery, Chonnam National University Medical School and Hospital, Gwangju 61469, Korea; my-love3835@hanmail.net

**Keywords:** ankylosing spondylitis, spinal fracture, spontaneous, spinal anesthesia

## Abstract

Factures in ankylosing spondylitis (AS) patients tend to occur due to the absence of motion between vertebrae, poor bone quality, and a long lever arm that generates extension force. However, most patients have a history of at least minor trauma. The aim of this report was that a vertebral fracture in a patient with AS can be caused not only by minor trauma, but also by position changes or maintenance of position for examination due to structural weakness. A 75-year-old woman with AS visited her local hospital on foot for back pain. She usually had back pain. However, she had increased back pain after falling over three weeks prior. In plain radiographs, no fracture was apparent. The doctor tried to perform magnetic resonance imaging (MRI) for further evaluation. However, several attempts of MRI failed due to continuous movement arising from pain. As a result, MRI was performed under spinal anesthesia for pain control. However, complete paraplegia developed during the MRI examination. MRI showed extension-type vertebral fracture with displacement and the patient was transferred to our hospital. We performed emergency posterior fusion, but neurological symptoms did not improve. This case suggests the need for careful positioning, sedation, or anesthesia when performing an examination or surgery in AS patients. We recommend that all patients with AS should be carefully positioned at all times during testing or surgery.

## 1. Introduction

Ankylosing spondylitis is a seronegative spondyloarthritis that mainly affects the axial skeleton and is characterized by the union of the sacroiliac joint and the ossification of soft tissue around the vertebral bodies. Fractures in ankylosing spondylitis tend to occur due to the absence of motion between vertebrae, poor bone quality, and a long lever arm that generates extension force. However, most patients have a history of at least minor trauma. We report a case of fracture in an ankylosing spondylitis patient associated with imaging examination and review the relevant literature.

## 2. Case Presentation

**Figure 1:**
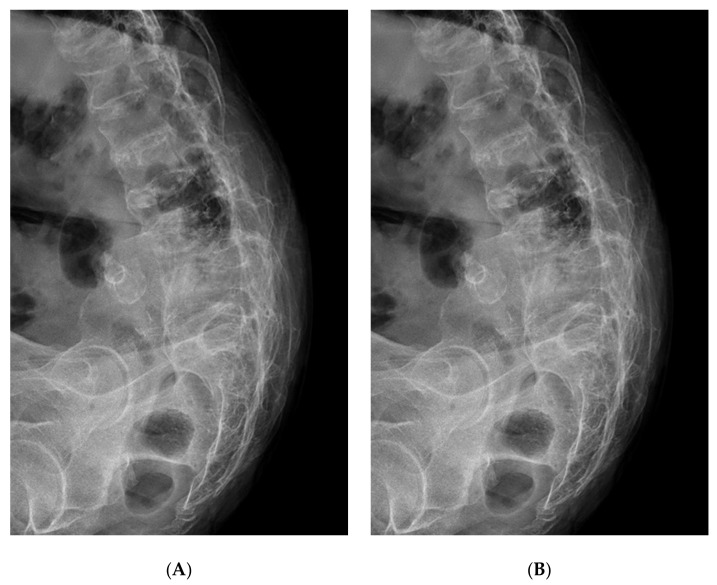
Flexion (**A**)-extension (**B**) lateral plain radiograph prior to cord injury.

The patient was informed that we wanted to submit data from her case for publication, and provided her consent. A 75-year-old woman with AS visited her local hospital on foot for back pain. She had had back pain for a long time. However, she experienced increased back pain after fell down three weeks prior. Plain radiographs showed a “bamboo spine” with generalized vertebral fusion due to AS, but no fracture was apparent (Figure 1). The pain did not improve and the doctor at the local hospital tried to perform magnetic resonance imaging (MRI) for further evaluation. However, several attempts failed due to continuous movement arising from pain and an inability to maintain the supine position. They tried again the next day and again failed. As a result, MRI was performed under spinal anesthesia for pain control.

Under monitoring, the patient took the right lateral decubitus position. The flexed spine position was not performed due to AS and pain. Using a paramedian approach, the needle was inserted through the L4–5 interlaminar space, and after confirming the outflow of CSF, 10 mg of 0.5% bupivacaine was injected. After observing that there was no abnormality in the patient for about 15 min, she was moved to the MRI room. She felt something breaking during the examination and at the same time felt a change in sensation. An acute fracture with displacement was found on the MRI. Before the examination, the neurological status of the lower extremity was normal. However, even after the spinal anesthesia was released, she continued to have complete paraplegia with no motor activity or sensation below the fracture site and she was transferred to our hospital.

**Figure 2:**
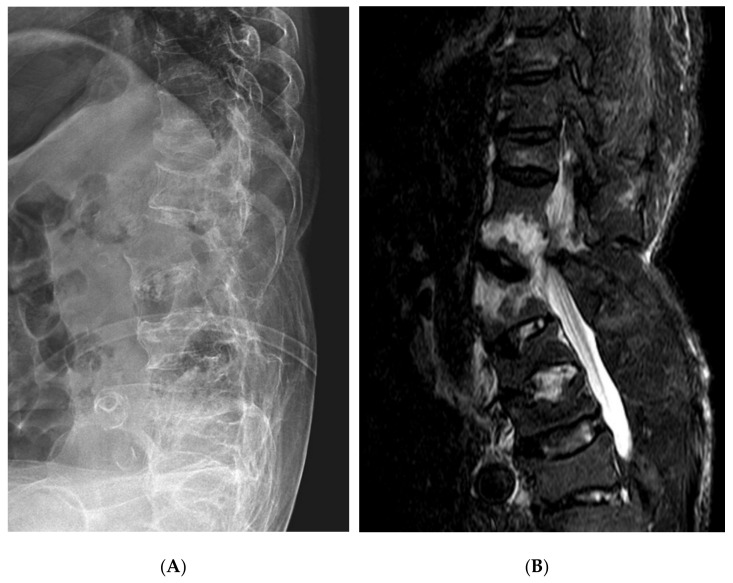
The lumbar spine lateral plain radiograph (**A**) and MRI sagittal view (**B**) after fracture.

Plain radiographs after admission to our hospital revealed a loss of previous thoracolumbar kyphosis with bamboo spine findings, and MRI showed fractures of T12 and L1 vertebrae (Figure 2). In a comparison with prior plain radiographs, an extension-type fracture with displacement was present. Osteopenia [T-scores: L1–4, −1.5 (0.831 g/cm^2^); femoral neck, −1.3 (0.665 g/cm^2^)] was observed on testing nine months before the injury.

**Figure 3:**
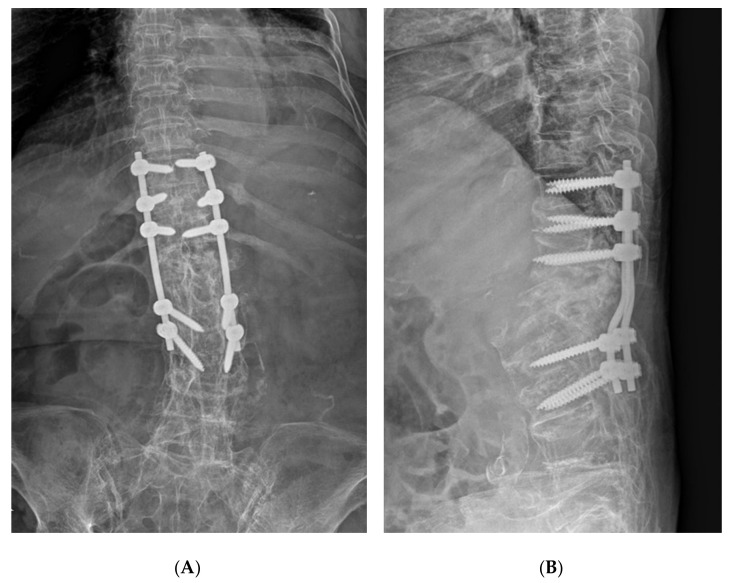
Postoperative plain radiograph (**A**,**B**).

Emergency posterior fusion was planned for the thoracolumbar fractures with complete paraplegia. We expected that fracture reduction would not be easy because of AS. To reduce displacement of the fracture, after general anesthesia, we tried some fracture reduction using abdominal support and operating table bending with the patient in the prone position before incision, but there was no change in the fracture site. Bone reduction was attempted again after incision and muscle dissection, but the fracture was not reduced at all. We performed in situ posterior fixation and fusion of T10–L3 vertebrae due to reduction failure and massive bleeding in the operative field (Figure 3). Upon imaging examination 10 months post operation, bone union was confirmed but neurological symptoms did not improve.

## 3. Discussion

Ankylosing spondylitis is a genetic disorder that causes ossification of the soft tissues around the sacroiliac joints and vertebral bodies. Fractures tend to occur due to absence of motion between vertebrae, poor bone quality, and a long lever arm that generates extension force. The reported incidence rate of fractures is 5–15%, and neurological complications such as cord injuries occur in about two-thirds of cases; the mortality rate is 18–32%. Vertebral fractures in AS occur most commonly in the cervical spine [1,2]. Fractures are thought to occur because of vertebral union with reduced capacity to absorb shock. Additionally, fractures are caused by the hyperextension mechanism in 75% of patients, which is vulnerable at the moment of extension due to a previous kyphotic deformity and long lever arm to generate extension force. The incidence of osteoporosis or osteopenia is higher in AS than in the general population [3,4,5]. Trent et al. noted that supine position is associated with a high risk of fracture in patients with AS. The separate weights of the cranial and caudal fused vertebral bodies can act as long lever arms on the upper and lower portions of the rigid kyphotic spinal column [6].

Our patient had experienced trauma three weeks prior when she fell down, but no fracture was apparent in plain radiographs. Because paraplegia developed during MRI examination, it is thought that a fracture was caused by the loss of lower body muscular tone under spinal anesthesia and extension force for kyphotic spine in the supine position. Of course, there might have been an unrecognized fracture that was not found in previous plain radiographs. However, whether or not there was a previous fracture, this illustrates a more important lesson that we have to be more careful in this situation. In retrospect, screening computed tomography (CT) should have been performed because the examination time was short and did not require anesthesia. However, it seems that the medical staff chose MRI examination rather than CT at the time because fractures were not found on plain radiographs and the trauma force from the fall three weeks prior was not significant. Additionally, in order to compensate for the loss of muscle tone due to spinal anesthesia and stress from the kyphotic spinal deformity of the patient, it was necessary to support the head, shoulders, pelvis, and legs with a blanket or pillow in the supine position during the MRI examination. Another option was that the imaging examination could have been performed in the lateral decubitus position from the beginning. Finally, the application of spinal anesthesia in a patient with AS should have been considered more carefully. The occurrence of fractures after spinal anesthesia is not a common complication of spinal anesthesia. However, in patients with severe spinal deformity (especially AS patients), the possibility of fractures due to loss of muscle tone after spinal anesthesia should be considered.

In 2006, Ruf et al. reported a cervical spine fracture due to a hyperextension injury during spinal surgery in two patients with AS [3]. In 2008, Danish et al. reported paraplegia during total hip arthroplasty in the supine position in two obese patients with AS [4]. In 2017, Maarouf et al. reported iatrogenic spinal cord injury in a patient with AS resulting from the use of rigid backboard immobilization during transport to the hospital after a traffic accident. The rigid spinal collar and board immobilization acted as an extension force on vertebrae with reduced flexibility [7]. Additionally, spontaneous fracture was reported at the C2 base in two cases [8,9] and the L4 body in one case [10]. However, the status of the spine at the time of injury was not described in detail. A search of the English language literature found no other reports of spontaneous fracture in patients with AS. Oh et al. reported a thoracic fracture following minor trauma in an elderly patient with AS associated with osteoporotic kyphosis, leading to complete paraplegia due to further worsening of the fracture-dislocation during a CT scan (Table 1) [11]. Our case differs from those previously reported in that spontaneous spine fracture occurred during imaging examination without a previously recognized fracture. This suggests the need for careful positioning, sedation, or anesthesia when performing an examination in the AS patient.

Most vertebral fractures in AS are very unstable and occur across all three columns. In addition, because of spinal fusion, the lever arm is increased and it is difficult or impossible to reduce the fracture when the fracture is displaced. In particular, these fractures occur mainly at the apex of kyphosis and the thoracolumbar junction. In these cases, reduction was more difficult due to shear forces on the thoracic vertebrae [6]. In our case, displacement between the T12 and L1 vertebrae was attempted for reduction. However, it was impossible to reduce the long rigid upper and lower vertebral column, leading to the need for in situ fixation.

## 4. Conclusions

Vertebral fracture in a patient with AS can be caused or aggravated not only by minor trauma, but also by position changes or maintenance of positions for examination due to structural weakness. Therefore, we recommend that all patients with AS should be carefully positioned at all times during testing or surgery. In particular, elderly patients with AS who are most likely to have osteoporosis should be made aware of the possibility of unexpected fracture.

## Figures and Tables

**Table 1:** Literature review regarding abnormal spine injury in ankylosing spondylitis.

Authors	Year	Number of Case	Level	Injury Mechanism	Symptom	Treatment	Remarks
Rapp GF et al. [10]	1974	1	L4	Spontaneous	Back pain	Posterior fusion	During bed rest
Gartman JJ Jr et al. [9]	1991	1	C2	Spontaneous	Radiating pain	Posterior decompression	Spontaneous neck spasm
Kremer P et al. [8]	1995	1	C2	Unknown	Tetraparesis	Unknown	Unknown
Ruf et al. [3]	2008	2	C6-7 C6-7	Fracture & dislocation Hyperextension injury	Quadriplegia Unknown	Posterior decompression Posterior fusion	Unknown During AS * lumbar surgery
Danish et al. [4]	2008	2	T11-12 T11	Hyperextension injury Hyperextension injury	Paraplegia Paraplegia	Posterior fusion Posterior decompression	During THA ^†^ in obese patients During THA ^†^ in obese patients
Oh JS et al. [11]	2016	1	T9-10	Hyperextension injury	Paraplegia	Immobilization	During CT ^#^ evaluation
Maarouf et al. [7]	2017	1	T10-11	Extension injury	Paraplegia	Spinal fusion	Rigid backboard immobilization

* Ankylosing spondylitis, ^†^ Total hip arthroplasty, ^#^ Computerized tomography.

## Data Availability

Data is contained within the article.
